# Magnitude of extended-spectrum *β*-lactamase and carbapenemase producing *Enterobacteriaceae* among commonly vended street foods in Arba Minch town, southern Ethiopia

**DOI:** 10.1186/s12866-023-03137-9

**Published:** 2023-12-08

**Authors:** Dagninet Alelign, Aschalew Kidanewold

**Affiliations:** Department of Medical Laboratory Sciences, Arba Minch University, Arba Minch, Ethiopia

**Keywords:** Extended-spectrum β-lactamase, *Enterobacteriaceae*, Street-vended food

## Abstract

**Background:**

The rising prevalence of extended-spectrum beta-lactamase and carbapenemase-producing *Enterobacteriaceae* (ESβL-PE) in street foods poses a significant risk to human health due to its epidemiological significance. Thus, the aim of this study was to determine the magnitude of foodborne *Enterobacteriaceae* that produce carbapenemase and ESβL, as well as their patterns of antibiotic resistance, in the studied area.

**Methods:**

A community-based cross-sectional study was carried out from January 1st, 2023, to February 30th, 2023. One hundred randomly chosen street-vended food items (one hundred grams of each food item) were aseptically collected, and aliquots of 0.1 ml from the homogenized (25 g of samples into 225 ml of buffered peptone water (BPW)) were inoculated on MacConkey agar and Xylose Lysine Deoxycholate Agar (XLD). *Enterobacteriaceae* isolates were identified using various biochemical tests. ESβL and carbapenemase were first screened by indicator cephalosporins and carbapenem antibiotics, respectively. ESβL and carbapenemase were confirmed by a double-disc synergy test and modified carbapenem inactivation methods, respectively. Kirby-Bauer disc diffusion method was used for the antimicrobial-resistant test.

**Results:**

A total of 112 *Enterobacteriaceae* belonging to six different genera were isolated. *E. coli was* attributed 39 (34.8%), followed by *Citrobacter* spp. 22 (19.6%) and *K. pneumoniae* 18 (16.1%), with only 8 (7.1%) isolated *Salmonella* spp. About 15.2% (*n* = 17) and 8.9% (*n* = 10) of *Enterobacteriaceae* were phenotypically confirmed to be extended-spectrum beta-lactamase (ESβL) and carbapenemase producers, respectively. The highest percentage of ESβL-producing isolates was attributed to *K. pneumoniae (n = 5), E. coli* (*n* = 4), and *Enterobacter* spp. (*n* = 3). *Proteus* spp. and *Salmonella* spp. isolates were carbapenemase-negative. All carbapenemase-positive isolates were found to be ESβL-producers. 70.6% (12/17) of ESβL-producing *Enterobacteriaceae were found to be* multidrug-resistant (MDR).

**Conclusion:**

A considerable number of multidrug-resistant ESβL and carbapenemase-producing *Enterobacteriaceae* were identified, suggesting that street foods may be a potential source of MDR foodborne infections. Consequently, it is important to conduct routine examinations of street food items and track trends in medication resistance.

## Introduction

Foodborne diseases caused by microbial pathogens are an essential contributor to morbidity and mortality, and food safety is rapidly becoming a major public health issue [1–3]. The global burden of foodborne diseases is estimated to be over 600 million, with 420,000 people dying each year [3]. A substantial portion is attributed to countries with a high prevalence of street-vended food, which accounts for up to 70% of all verified outbreaks [4, 5]. In Ethiopia, where street food is common, food-borne-associated diarrheal sickness is the second leading cause of death, following typhoid fever [5, 6].

Despite their many benefits, street-vended foods pose a significant public health risk as a potential vehicle for foodborne pathogens during preparation, post-cooking, and other handling stages and have been identified as major reservoirs for various antibiotic-resistant pathogens and extended-spectrum beta-lactamases (ESβL) producers’ bacteria in the community setting [5–7]. As a result, travelers’ diarrhea caused by eating street food is considered to be a major discomfort for visitors arriving in low-income nations and may be a significant barrier to tourism [6, 8].

The rise of ESβL-producing foodborne Enterobacteriaceae is becoming a challenging public health problem for the food supply chain, and the contribution of the food chain to the occurrence of ESβL bacteria has a large influence on its emergence and spread in the general population, decreasing therapeutic options, increasing mortality, and necessitating an extended hospital stay [9–11]. β-lactamase and carbapenemase production ability is one of the most important mechanisms of resistance displayed by the *Enterobacteriaceae* that can cleave the β-lactam ring of β-lactam antibiotics and confer increased resistance to commonly used and newer β-lactam antibiotics, including third- and fourth-generation cephalosporins and monobactams, whereas carbapenemases are enzymes that are able to hydrolyze nearly all β-lactamase antibiotics, including carbapenems [12, 13].

Consequently, studies on the microbial quality of various ready-to-eat foods vended on the street in low-income countries, including Ethiopia, revealed that more than 60% of contaminants were Gram-negative bacilli with a higher rank of *Enterobacteriaceae* and a high rate of resistance to commonly prescribed antibiotics, posing a significant risk due to their epidemiological significance [5, 6, 14].

However, the prevalence of ESβL and/or carbapenemase-producing *Enterobacteriaceae* in the study area among street-vended foods has not yet been explored. While in Arba Minch town, it is usual to observe roadside ready-to-eat (RTE) foods being sold in an irregular manner. Therefore, the purpose of the study was to determine the potential burden of *Enterobacteriaceae* that produce carbapenem and/or ESβL that contaminate RTE street-vended food in Arba Minch, southern Ethiopia.

## Materials and methods

### Study design, study area and period

A community-based cross-sectional study was conducted from January 1st, 2023, to February 30th, 2023, in regular street food vending areas of Arba Minch town, namely: Sikela (nearby bus station), Shecha (nearby Gebeya Dar), and Konso Sefer (nearby Arba Minch General Hospital). Various ready-to-eat street foods are offered on the streets of the town. Arba Minch is the administrative center for the Gamo Zone, which is located in southern Ethiopia, 454 km from Addis Ababa. The city of Arba Minch and its surroundings provide a variety of unique and memorable tourist attractions, and unlike foods in hotels and resorts, the majority of visitors choose street-vended fast food.

### Sample collection, culture and identification

A total of 100 ready-to-eat street-vended food samples (100 g from each food item) from four different types of frequently vended and highly consumed street foods, such as “Sambusa” (*n* = 25), “Ambasha” (*n* = 25), “Potato Chips” (*n* = 25), and “Koker” (*n* = 25), were collected by well-trained sample collectors aseptically using sterile aluminium foil and transported using an icebox within 3–5 °C. Within one hour of being collected, the samples were sliced into smaller pieces with a sterile surgical blade and weighed at 25 g on an electronic digital scale. The measured 25 g samples were homogenised in 225 mL of buffered peptone water (BPW) [Oxoid, Hampshire, UK]. 0.1 mL aliquots from the homogenised or mixed tube were distributed onto MacConkey Agar plates [HiMedia Laboratory Pvt. Ltd., Mumbai, India] and Xylose Lysine Deoxycholate Agar (XLD) [HiMedia Laboratory Pvt. Ltd., Mumbai, India] with an L-shaped sterile wire spreader and incubated at 35 °C for 24 h [6, 14, 15].

*Enterobacteriaceae* were identified as pink to reddish-purple colonies on MacConkey Agar plates with or without precipitate haloes, while a black colony surrounded by a red colour on XLD was considered to be *Salmonella* spp. Cell morphology, Gram staining, and various biochemical tests [HiMedia Laboratory Pvt. Ltd., Mumbai, India] were used to characterise the isolated pure colonies of *Enterobacteriaceae* to the genus and species level, including Triple Sugar Iron Agar (TSI), Simmon Citrate, Indole, Motility, Methyl Red-Voges Proskauer (MR-VP), and Urease, Oxidase, Gas, and H_2_S production. The pure isolate was subcultured onto nutrient agar plates for confirmatory assays of antibiotic susceptibility, ESβL, and carbapenemase production [15–17].

### Screening of ESβL and Carbapenemase-producing Enterobacteriacae

ESβL-producing *Enterobacteriaceae* were first screened for ESβL-production by indicator cephalosporins (cefotaxime (30 μg) and ceftazidime (30 μg)). Isolates that showed an inhibition zone size of ≤27 mm with cefotaxime (30 μg) ≤ 22 mm and/or with ceftazidime (30 μg) were considered potential ESβL-producers and were selected for confirmation for ESβL-production using the Double-Disc Synergy Test (DDST), whereas isolates with reduced susceptibility to meropenem (diameter of the zone of inhibition ≤13 mm), as demonstrated by the disc diffusion method according to the CLSI 2020 guidelines, were considered carbapenem-resistant strains and were selected for confirmation with the Modified Carbapenem Inactivation Method (mCIM) [13, 18–20].

### Phenotypic confirmation of ESβL by double-disc synergy test (DDST)

Antibiotic discs containing cephalosporins (cefotaxime (30 μg) and ceftazidime (30 μg)) were applied to MHA plate inoculated with a bacterial suspension of 0.5 McFarland standard next to a disc with amoxicillin-clavulanic acid and incubated overnight (18–24 h) at 37 °C. The distance between the discs, 20 mm from centre-to-centre, is optimal for cephalosporin 30 μg discs. The distance between the discs however will be reduced (15 mm) or expanded (30 mm) for strains with very high or low levels of resistance, respectively. A positive result was indicated when the inhibition zones around any of the cephalosporin discs were enhanced or there was a “keyhole” or ≥ 5 mm increase in zone diameter in the direction of the disc of amoxicillin-clavulanic acid [13, 18–20].

### Modified Carbapenem inactivation method (mCIM)

A loopful of test isolates of the respective species of *Enterobacteriaceae* (1 μl) was suspended in 2 mL of tryptic soy broth [Oxoid Ltd., Basingstoke, United Kingdom], and a meropenem disc was immersed in the suspension and incubated for a minimum of 4 h at 37 °C. A 0.5 McFarland suspension of *E. coli* ATCC 25922 indicator strains was prepared in normal saline using the direct colony suspension method and inoculated on an MHA plate using the routine disc diffusion procedure. Then the meropenem disc was removed from the TSB and placed on an MHA plate inoculated with *E. coli* ATCC 25922 and incubated at 37 °C for 24 h. If the isolate produces carbapenemase, the meropenem in the disc is hydrolyzed, and there is either no zone of inhibition or only limited enhancement in the inhibition zone corresponding to the meropenem-susceptible *E. coli* (ATCC 25922). Thus, results were judged to be positive for inhibition zones with a diameter of ≤15 mm or presence of pinpoint colonies within a 16–18 mm, and negative for ≥19 mm [18–20].

### Antibiotic susceptibility testing

Antimicrobial susceptibility testing was carried out by the Kirby-Bauer disc diffusion method [21], and the results or the diameter of the zone of inhibition were expressed as susceptible, intermediate, or resistant according to the CLSI 2020 guideline after overnight incubation at 37 °C. After preparation of 0.5 McFarland turbidity inoculums, Muller-Hinton Agar (MHA) [Oxoid LTD, Basingstoke, Hampshire, and United England] plates were inoculated, and antimicrobial discs were applied to the plate. The antibiotic discs [Abtek Biologicals Ltd., Liverpool, UK] used in this study were ampicillin (10 μg), amoxicillin-clavulanic acid (20/10 μg), cefotaxime (30 μg), meropenem (10 μg), gentamicin (10 μg), azithromycin (15 μg), tetracycline (30 μg), ciprofloxacin (5 μg), sulfamethoxazole-trimethoprim (3.75/1.25 μg), and chloramphenicol (30 μg). An *Enterobacteriaceae* isolate was considered multidrug-resistant (MDR) if it was non-susceptible to at least one antimicrobial drug in three or more different classes or groups of antibiotics [19].

### Data quality assurance

Reference strains (known for producing ESBL and carbapenemase) obtained from the Ethiopian public health institution (EPHI): *E. coli* ATCC 25922 and *K. pneumoniae* ATCC 700603, were used to check the quality of ESBL and carbapenemase test procedures, the quality of culture media, biochemical tests, and the effectiveness of antibiotic discs. Standard operating procedures (SOP) were strictly implemented from sample collection up to final microbiological identification. All culture media were prepared following the manufacturer’s instructions, and the sterility of the culture media was tested by incubating 5% of the batch at 35–37 °C overnight for evaluation of possible contamination.

## Results

### Prevalence of *Enterobacteriaceae* isolates

**Fig. 1:**
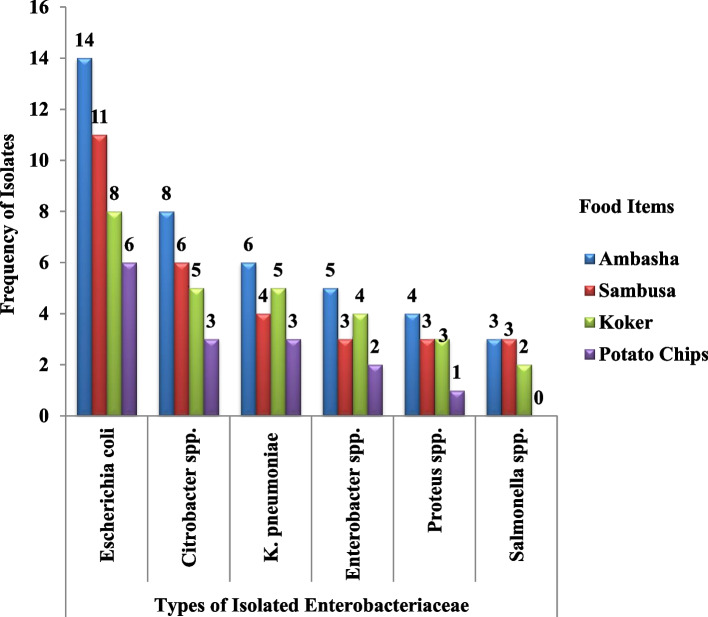
Types of *Enterobacteriaceae* Isolated from Commonly Vended Street Food Items in Arba Minch Town, Southern Ethiopia, 2023

A total of 112 *Enterobacteriaceae* belonging to six different genera were isolated from 100 different commonly vended street food samples and identified using biochemical characteristics, and their production of ESβL and/or carbapenemase was subsequently validated. 40 (35.7%) of the total isolates were found in “Ambasha,” followed by 30 (26.8%) from “Sambusa,” and 15 (13.4%) of the *Enterobacteriaceae* isolates were recovered from “Potato Chips.” The most prevalent isolate was *E. coli*, which accounted for 39 (34.8%), followed by *Citrobacter* spp. 22 (19.6%) and *K. pneumonia*e 18 (16.1%), with only 8 (7.1%) isolated *Salmonella* spp. (Fig. 1).

### ESβL and Carbapenemase producing *Enterobacteriaceae* isolates

In this study, among the overall 38 (33.9%) and 15 (13.4%) *Enterobacteriaceae* suspected to be ESβL and carbapenemase-producers, 17 (15.2%) and 10 (8.9%) were phenotypically confirmed, respectively, to be ESβL and carbapenemase producers. *K. pneumoniae (n = 5)* had the highest percentage of ESβL-producing isolates, followed by *E. coli* (*n* = 4) and *Enterobacter* spp. (*n* = 3), while four *K. pneumoniae*, three *E. coli*, two *Citrobacter* spp., and one *Enterobacter* spp. isolates were found to be carbapenemase producers. However, carbapenemase production was not seen in isolated *Proteus* spp. or *Salmonella* spp. On the other hand, all carbapenemase-positive *Enterobacteriaceae* isolates were found to be ESβL-producers (Table 1).

**Table 1:** Phenotypically Suspected and Confirmed ESβL and Carbapenemase-Producing *Enterobacteriaceae* Isolates from Commonly Vended Street Foods in Arba Minch Town, Southern Ethiopia, 2023

Types of Isolates	No. (%) of ESβL and Carbapenemase-producing *Enterobacteriaceae* (*n* = 112)			
	ESβL suspected	DDST confirmed	Carbapenemase suspected	mCIM confirmed
***E. coli*** **(*n* = 39)**	11 (28.2)	4 (10.2)	4 (10.2)	3 (7.7)
***Citrobacter*** **spp. (*n* = 22)**	6 (22.3)	2 (9.1)	3 (13.6)	2 (9.1)
***K. pneumoniae*** **(*n* = 18)**	10 (55.6)	5 (27.8)	4 (22.2)	4 (22.2)
***Enterobacter*** **spp. (*n* = 14)**	5 (35.7)	3 (21.4)	2 (14.3)	1 (7.1)
***Proteus*** **spp. (*n* = 11)**	4 (36.4)	2 (18.2)	1 (9.1)	0 (0)
***Salmonella*** **spp. (*n* = 8)**	2 (25)	1 (12.5)	1 (12.5)	0 (0)
**Total (*n* = 112)**	38 (33.9)	17 (15.2)	15 (13.4)	10 (8.9)

*Abbreviations:* *ESβL* Extended-spectrum Beta-lactamase, *DDST* Double-Disc Synergy Test, *mCIM* Modified Carbapenem Inactivation Method, *spp* Species

### Antimicrobial resistance patterns of ESβL-producing *Enterobacteriaceae*

The antibiogram testing findings revealed that 88.2% (15/17) of isolated ESβL-producing *Enterobacteriaceae (ESβL-PE)* were found to be resistant to ampicillin and tetracycline, whereas 64.7% (11/17) were resistant to ciprofloxacin, sulfamethoxazole-trimethoprim, and chloramphenicol. Moreover, 3.5% (6/17) and 4.7% (8/17) of ESβL-PE were found to be amoxicillin-clavulanic acid and gentamicin-resistant, respectively, the highest percentage accounting for *E. coli* and *K. pneumoniae isolates.* One ESβL-producing *Salmonella* spp. showed ciprofloxacin resistance, which is the most prescribed antibiotic in the setting for the treatment of *Salmonella* infection (Table 2).

**Table 2:** Antimicrobial Resistance Patterns of ESβL-Producing *Enterobacteriaceae* Isolates from Commonly Vended Street Foods in Arba Minch Town, Southern Ethiopia, 2023

Tested Antibiotics	No. (%) of Resistant Isolates Respective to Each Tested Antibiotics							
	AMC	AM-CL	GEN	AZM	TET	CPR	SXT	CHL
*E. coli* (*n* = 4)	4 (100)	2 (50)	2 (50)	3 (75)	4 (100)	3 (75)	3 (75)	3 (75)
*Citrobacter* spp. (*n* = 2)	1 (50)	1 (50)	1 (50)	1 (50)	1 (50)	1 (50)	1 (50)	1 (50)
*K. pneumoniae* (*n* = 5)	4 (80)	2 (50)	3 (60)	3 (60)	4 (80)	3 (60)	4 (80)	3 (60)
*Enterobacter* spp. (*n* = 3)	3 (100)	1 (33.3)	1 (33.3)	2 (66.7)	3 (100)	2 (66.7)	2 (66.7)	2 (66.7)
*Proteus* spp. (*n* = 2)	2 (100)	0 (0)	1 (50)	1 (50)	2 (100)	1 (50)	1 (50)	1 (50)
*Salmonella* spp. (*n* = 1)	1 (100)	0 (0)	0 (0)	0 (0)	1 (100)	1 (100)	0 (0)	1 (100)
**Total (*n* = 17)**	15 (88.2)	6 (3.5)	8 (4.7)	10 (58.8)	15 (88.2)	11 (64.7)	11 (64.7)	11 (64.7)

*Abbreviations:* *AMC* ampicillin, *AM-CL* amoxicillin-clavulanic acid, *GEN* gentamicin, *AZM* azithromycin, *TET* tetracycline, *CPR* ciprofloxacin, *SXT* sulfamethoxazole-trimethoprim, *CHL* chloramphenicol, *spp* species

### Multidrug-resistant profile of ESβL-producing *Enterobacteriaceae*

The overall prevalence of multidrug-resistant (MDR) ESβL-PE in this study was 70.6% (12/17). The most prevalent MDR ESβL-PE isolates were *K. pneumonia* (*n* = 4), *E. coli* (*n* = 3), and *Enterobacter* spp. (*n* = 2). Only one of each of the isolated ESβL-producing *Citrobacter* spp., *Proteus* spp., and *Salmonella* spp. was found to be MDR. The majority, 47.1% (8/17), of MDR-ESβL-producing *Enterobacteriaceae* were resistant to three drug classes (Table 3).

**Table 3:** Multidrug-Resistant Profile of ESβL-Producing *Enterobacteriaceae* Isolates from Commonly Vended Street Foods in Arba Minch Town, Southern Ethiopia, 2023

Bacterial Isolates	Antibiogram Pattern (%)			
	R3	R4	≥ R5	Total
***E. coli*** **(*n* = 4)**	2 (50)	1 (25)	0 (0)	3 (75)
***Citrobacter*** **spp. (*n* = 2)**	1 (50)	0 (0)	0 (0)	1 (50)
***K. pneumoniae*** **(*n* = 5)**	2 (40)	1 (20)	1 (20)	4 (80)
***Enterobacter*** **spp. (*n* = 3)**	1 (33.3)	1 (33.3)	0 (0)	2 (66.7)
***Proteus*** **spp. (*n* = 2)**	1 (50)	0 (0)	0 (0)	1 (50)
***Salmonella*** **spp. (*n* = 1)**	1 (50)	0 (0)	0 (0)	1 (50)
**Total MDR isolates (*n* = 17)**	8 (47.1)	3 (17.6)	1 (5.9)	12 (70.6)

Where: R3, R4, and R5: Resistant to at least one antibiotic from each of the three classes, four classes, and five or more classes of antibiotics, respectively

*Abbreviations:* *MDR* Multidrug-Resistant, *spp* Species

## Discussion

The global prevalence of community-acquired ESβL and/or carbapenemase-producing *Enterobacteriaceae* has increased, affecting food product chains. In this investigation, 112 *Enterobacteriaceae* isolates were found in 100 different street vendor food samples. The most common isolates were *E. coli* 39 (34.8%), *Citrobacter* spp. 22 (19.6%), and *K. pneumoniae* 18 (16.1%). The prevalence of *Enterobacteriaceae* in this study coincides with recent investigations of various ready-to-eat street items and fresh vegetables [13, 22]. The high *Enterobacteriaceae* contamination could be due to post-processing exposure as well as inadequate sanitation among vendors and their vending surroundings [6, 13].

The prevalence of phenotypically confirmed ESβL-PE isolates was found to be 15.2% (*n* = 17) with a 95% CI of 8.9–22.3, which is in line with previous studies conducted among different street food items in China (11.1%) [23], from Spain (17.8%) [24], and in Delhi and Bareilly cities (19.7%) [25], as well as a report from a systematic review and meta-analysis done in Ethiopia (18%) [26]. However, the overall ESβL confirmed in this study was lower than reports from Mangalore, India (25.42%) [13], Tamale, Ghana, at 65.5% [27], and India, Thailand, and Vietnam (25.4%) [22]. The presence of ESβL in street food items could be related to unsanitary practises in food production, storage, and handling. Although previously there were no study reports about carbapenemase-producers, *Enterobacteriaceae*, from street-vended foods, in this study 10 (8.9%) of isolated *Enterobacteriaceae* were phenotypically confirmed carbapenemase producers, and all carbapenemase producers were found to be ESβL-producers. Carbapenems are the medication of choice for ESβL -producing pathogens; however resistance to them leaves treatment options with no obvious alternative [13, 28]. The presence of *E. coli* and *K. pneumoniae* may contribute to the high percentage of ESβL and/or carbapenemase-positive isolates, indicating food contamination and a high presence and rate of gene transfer [13, 25].

This study reports that 29.4% (5/17) of ESβL producers were *K. pneumoniae* and 23.5% (4/17) were *E. coli*, which is in agreement with studies done on different ready-to-eat street food samples in Tamale, Ghana. ESβLs were detected in approximately 55.0% of the *E. coli* strains and close to 45.0% in *Salmonella* spp. [27], and studies in the Philippines [8] report that ESβL-producers were *E. coli* (80%), followed by *Enterobacter* spp. (9.1%), and *K. pneumoniae* (3.6%). Likewise, a study from South Korea [29] has noted 15.8% of ESβL-positive *E. coli* and 84.2% of ESβL-producing *K. pneumoniae*, while reports from Mangalore city in India [13] from ready-to-eat street foods report that 18.51% of *E. coli* and 34.37% of *K. pneumoniae* were phenotypically confirmed to be ESβL-producers. Variations in street-vended food type and ingredients, preparation procedures, personal hygiene, vendor hygienic practises, and serving practises, as well as a diversity of surroundings and climatic circumstances, are chiefly responsible for these disparities. High ESβL-PE counts, on the other hand, could be ascribed to post-processing contamination as well as poor sanitary conditions among vendors and their vending surroundings.

The present study reports a high prevalence of multidrug-resistant (MDR) ESβL–PE 70.6% (12/17); the majority, 47.1% (8/17), of MDR ESβL-PE were resistant to three drug classes. About 88.2% of isolated ESβL-PE were found to be resistant to ampicillin and tetracycline, while amoxicillin-clavulanic acid and gentamicin-resistant ESβL-PE were found to be 3.5 and 4.7%, respectively. This might be due to the fact that ESβL -PE can display co-resistance to non-beta-lactam antibiotics, which is in line with previous studies [13, 28]. However, this finding is lower than reports from Tamale, Ghana (100%) [27], India, Thailand, and Vietnam (78.3%) [22], and in Delhi and Bareilly cities (85.71%) [25]. Such variations in drug resistance patterns may be attributed to differences in irrational antibiotic use and suggest appropriate measures be taken to control the indiscriminate use of pathogens with resistant genes, such as avoiding antibiotics without a prescription for treatments and improving sanitation and hygiene standards for RTE street foods through food handling procedures and food safety practises [13, 22].

### Limitation of the study

Results might not apply to other sites because of the small sample size and restricted vending area used for sample collection. Furthermore, rather than using combined disc diffusion as an ESBL confirmatory test, the Double-Disc Synergy Test (DDST) was employed due to cost and resource limitations. Similarly, neither the molecular characterisation of ESβL-PE nor the impact of AmpC were carried out.

## Conclusion and recommendation

The overall prevalence rate of ESβL-PE (15.2%) was comparable with previous reports in the setting among different clinical and non-clinical specimens. The predominant isolates of ESβL-PE were *E. coli and K. pneumoniae*. ESβL-PE isolates showed a higher degree of resistance against commonly used antibiotics such as ampicillin, tetracycline, ciprofloxacin, sulfamethoxazole-trimethoprim, and chloramphenicol. Similarly, the overwhelming carbapenemase-producing *Enterobacteriaceae* (8.9%) is concerning because the bacteria developed the carbapenem resistance gene prior to usage in our setting, which requires immediate attention from all relevant parties. Moreover, 70.6% of isolated ESβL-PE was found to be Multidrug-resistant (MDR). In general, this study provides useful baseline data for public health professionals in the management of human infections caused by foodborne diseases in the surveyed area, and thus, continuous and regular inspection of regularly vended street food items, as well as environmental sanitation in the vending area and routine antibiotic resistance testing for foodborne bacterial infection, are required.

## Data Availability

The data underlying the study’s findings are available upon reasonable request from the corresponding author.

## Abbreviations

ATCC: American Type Culture Collection
BPW: Buffered Peptone Water
CLSI: Clinical and Laboratory Standards Institute
DDS: Double -Disc Synergy Test
ESβL–PE: Extended-Spectrum Beta-Lactamase-Producing *Enterobacteriaceae*
RTE: Ready-to-Eat Food
mCIM: Modified Carbapenem Inactivation Method
MDR: Multi Drug Resistant
